# Verzicht auf Strahlentherapie in Abhängigkeit vom Therapieansprechen bei Kindern und Jugendlichen mit mittlerer und weiter fortgeschrittener Hodgkin-Erkrankung. Vergleich mit anderen Konsolidierungstherapien (EuroNet-PHL-C1) – eine Titrationsstudie

**DOI:** 10.1007/s00066-022-01923-4

**Published:** 2022-04-01

**Authors:** Martin G. Sauer

**Affiliations:** Department of Pediatric Hematology and Oncology, Medizinische Hochschule Hannover, OE 6780, Carl-Neuberg-Straße 1, 30625 Hannover, Deutschland

## Fragestellung

Kinder und Jugendliche mit „intermediate-stage“ und „advanced-stage“ klassischem Hodgkin-Lymphom erreichen heute ein ereignisfreies Überleben (EFS) im Bereich von 90 %. Standardtherapie in Europa ist derzeit eine Induktion mit Vincristin, Etoposid, Prednison und Doxorubicin (OEPA), gefolgt von einer Konsolidierung mit Cyclophosphamid, Vincristin, Prednison und Procarbazin (COPP) bzw. Strahlentherapie. Die Spätfolgen sind jedoch erheblich. Darauf aufbauend sollte nun die Frage beantwortet werden, ob bei gutem Initialansprechen auf OEPA, gefolgt von modifizierter Konsolidierungschemotherapie, auch ohne Strahlentherapie die guten Überlebensraten erhalten und die Gonadotoxizität reduziert werden können.

## Methoden

An der Studie (2007–2013) nahmen 186 Zentren aus 16 europäischen Ländern teil. Alle Patienten wurden mit zwei Zyklen OEPA behandelt und im Anschluss das Therapieansprechen anhand Volumenreduktion (MRT) und metabolischem Ansprechen (PET) bestimmt (Stratifizierung). Bei gutem Ansprechen wurde im Rahmen der Konsolidierungstherapie auf die Bestrahlung verzichtet, ansonsten wurde sie nach Standard durchgeführt. Der Chemotherapieanteil der Konsolidierung wurde randomisiert, und zwar entweder mit konventionellem COPP oder COPDAC durchgeführt. Letzteres unterscheidet sich durch die Substitution von Procarbazin durch das weniger gonadotoxische Dacarbazin. Das Primärziel war die Erhaltung des guten EFS nach 5 Jahren bei Kindern, die aufgrund eines guten initialen Therapieansprechens keine Bestrahlung mehr erhielten. Gleichzeitig sollte eine Noninferiorität hinsichtlich des EFS in der COPDAC-Gruppe nachgewiesen werden. Insgesamt wurden 2102 Patienten in die Studie eingebracht.

## Ergebnisse

Insgesamt wurden 937 (69 %) Patienten randomisiert, und zwar 471 nach COPP und 466 in COPDAC. Die mediane Nachbeobachtung betrug 66,5 Monate. Von 1287 Protokollpatienten hatten 514 (40 %) ein gutes Therapieansprechen erreicht und erhielten deshalb keine Bestrahlung. 773 Kinder (60 %) erhielten eine Radiotherapie aufgrund eines unzureichenden Initialansprechens. Bei den Patienten, die aufgrund des guten Ansprechens keine Bestrahlung erhielten, wurde ein EFS nach 5 Jahren von 90,1 % erreicht. Dabei lag das EFS nach COPP bei 89,9 %, nach COPDAC bei 86,1 %.

## Schlussfolgerung der Autoren

Auf eine Radiotherapie kann bei gutem Therapieansprechen auf zwei Zyklen OEPA verzichtet werden, wenn sich eine Konsolidierung mit COPP oder COPDAC anschließt. COPDAC scheint dabei zwar etwas weniger effektiv als COPP zu sein, spart aber substanziell Gonadotoxizität ein. COPDAC wird deshalb zukünftig zum Therapiestandard in der Konsolidierung empfohlen werden.

## Kommentar

Kinder und Jugendliche, die nach der klassischen Therapie ein Hodgkin-Lymphom überlebt haben, entwickeln ein substanzielles Risiko für Zweitmalignome, Infertilität und frühzeitig einsetzende kardiovaskuläre Erkrankungen [1, 2]. Bei den ausgezeichneten Heilungsraten besteht die Herausforderung darin, die Gonadotoxizität (durch Procarbazin) und Spätnebenwirkungen durch Reduktion der Bestrahlung zu minimieren. Die Beurteilung des initialen Therapieansprechens mittels PET ist bei der Therapie des Hodgkin-Lymphoms mittlerweile weit verbreitet [3]. Darüber hinaus ist die Vermeidung von Procarbazin bekanntermaßen in seiner Wirksamkeit den Standardkombinationen vergleichbar, und zwar bei Mädchen und Jungs [4]. Die besprochenen Ergebnisse der EuroNet-PHL-C1-Studie zeigen nun eindrücklich, dass bei Kindern und Jugendlichen nach gutem initialem Therapieansprechen auf eine Strahlentherapie verzichtet werden kann, ohne die herausragenden Heilungsraten zu kompromittieren. Zusätzlich wird dabei der Therapieerfolg durch den Ersatz von COPP durch COPDAC in der Konsolidierung nicht gefährdet. Damit kann bei ca. 40 % aller Patienten mit „intermediate stage“ und „advanced stage“ auf die Bestrahlung verzichtet werden, was das Auftreten von Spätnebenwirkungen vermutlich deutlich reduzieren wird. Durch den Einsatz von Dacarbazin statt Procarbazin ist zusätzlich ein großer Gewinn beim Fertilitätserhalt zu erwarten.

## Fazit

Die Ergebnisse des „EuroNet-PHL-C1 trial“ werden den Therapiestandard des Hodgkin-Lymphoms in Europa bei Kindern und Jugendlichen neu setzen. Mit einem starken Einfluss der Ergebnisse auf andere internationale Konsortien ist zu rechnen.

*Martin G. Sauer, Hannover*
